# Access to institutional delivery care and reasons for home delivery in three districts of Tanzania

**DOI:** 10.1186/1475-9276-13-48

**Published:** 2014-06-16

**Authors:** Amon Exavery, Almamy Malick Kanté, Mustafa Njozi, Kassimu Tani, Henry V Doctor, Ahmed Hingora, James F Phillips

**Affiliations:** 1 Ifakara Health Institute, Plot 463, Kiko Avenue, Off Mwai Kibaki Road, Mikocheni, P.O. Box 78373, Dar es Salaam, Tanzania; 2 Department of Population and Family Health, Mailman School of Public Health, Columbia University, New York, USA; 3 Jhpiego, Dar es Salaam, Tanzania; 4 United Nations Office on Drugs and Crime, United Nations House, Abuja, Nigeria

**Keywords:** Access, Delivery care, Institutional delivery, Kilombero, Rufiji, Ulanga, Tanzania

## Abstract

**Introduction:**

Globally, health facility delivery is encouraged as a single most important strategy in preventing maternal and neonatal morbidity and mortality. However, access to facility-based delivery care remains low in many less developed countries. This study assesses facilitators and barriers to institutional delivery in three districts of Tanzania.

**Methods:**

Data come from a cross-sectional survey of random households on health behaviours and service utilization patterns among women and children aged less than 5 years. The survey was conducted in 2011 in Rufiji, Kilombero, and Ulanga districts of Tanzania, using a closed-ended questionnaire. This analysis focuses on 915 women of reproductive age who had given birth in the two years prior to the survey. Chi-square test was used to test for associations in the bivariate analysis and multivariate logistic regression was used to examine factors that influence institutional delivery.

**Results:**

Overall, 74.5% of the 915 women delivered at health facilities in the two years prior to the survey. Multivariate analysis showed that the better the quality of antenatal care (ANC) the higher the odds of institutional delivery. Similarly, better socioeconomic status was associated with an increase in the odds of institutional delivery. Women of *Sukuma* ethnic background were less likely to deliver at health facilities than others. Presence of couple discussion on family planning matters was associated with higher odds of institutional delivery.

**Conclusion:**

Institutional delivery in Rufiji, Kilombero, and Ulanga district of Tanzania is relatively high and significantly dependent on the quality of ANC, better socioeconomic status as well as between-partner communication about family planning. Therefore, improving the quality of ANC, socioeconomic empowerment as well as promoting and supporting inter-spousal discussion on family planning matters is likely to enhance institutional delivery. Programs should also target women from the *Sukuma* ethnic group towards universal access to institutional delivery care in the study area.

## Introduction

Virtually all the 287,000 annual deaths from pregnancy-related complications occur in developing regions, particularly in Africa and Asia [1,2]. Also more than 20 million women worldwide become pregnant annually, 15% of whom are likely to develop complications that will require attention of skilled obstetric care to prevent morbidity and mortality [3]. Haemorrhage, toxaemia, obstructed labour, and sepsis are universally documented as immediate causes of maternal deaths [3]. In addition, haemorrhage and hypertensive disorders are the leading causes of maternal mortality in developing countries [4]. Several other causes of maternal mortality reportedly exist [5,6]. However, most of the maternal deaths are preventable if deliveries were overseen by skilled personnel [7]. Also access to essential obstetric care would result in a 52% decline in the current burden of global maternal deaths [8]. Delivering at health facilities enables women receive proper medical attention and care during childbirth. This is fundamentally encouraged as a single most important strategy in preventing maternal and neonatal deaths [9]. However, rates of health facility delivery and deliveries that are attended by skilled providers remain low in many developing countries and are lowest in sub-Saharan Africa (SSA) [10]. Studies have shown that the average facility delivery in SSA in 2008 was only 47% and the maternal mortality ratio was highest in this region [10]. Although strategies like user-fee exemption for delivery and associated cares have been in place in many countries to enhance access, barriers still exist as universal coverage remains elusive.

In Tanzania, the health policy stipulates that maternal and child health services are exempted from user-fees in order to increase their uptake by poor and vulnerable groups [11]. However, some evidence shows that out-of-pocket spending still limits access to maternal and child health care in Tanzania [12]. Therefore, efforts are still needed to increase access to facility delivery care for all women. The Tanzania Demographic and Health Survey (TDHS) estimated that half of all deliveries in Tanzania in 2010 occurred at health facilities [13,14]. Intra-country variations exist, ranging from 24% in Pemba North to 90% in Dar es Salaam [13]. While it is clear that several factors may be attributing to the situation, wealth status may not be expected assuming that the user‒fee exemption policy was successful in making facility delivery care equally accessible between the poor and the rich. Otherwise, reforming universal coverage strategies may be necessary in order to make facility delivery care accessible to all population groups.

Earlier studies have documented several factors associated with place of delivery. A study from rural northern Ghana [15] found that relative to having had been seen by a doctor during antenatal care (ANC), women who were seen by a clinic nurse or midwife, community nurse, and a traditional birth attendant were more likely to deliver at home. The same study found further that while lack of access to ANC increased the odds of home delivery, as the number of times a women received ANC increased, the chances for home delivery declined remarkably. Ethnicity was also associated with home delivery in this study. In Dhading district of Nepal, one study found that maternal education, ethnicity, and parity were significantly associated with place of delivery [16]. Moreover, distance, socioeconomic status, parity, and ANC utilization were identified as significant predictors of place of delivery in Nepal [17]. A study from Nigeria shows that maternal education level, husband’s occupation, and age at first pregnancy were the main determinants of place of delivery [3]. In rural Tanzania, Mrisho and colleagues [18] found that lack of money, lack of transport, sudden onset of labour, short labour, staff attitudes, lack of privacy, socio-cultural beliefs and the pattern of decision-making power within the household were perceived as key determinants of the place of delivery. The study observed further that household headship, education, maternal age, and socioeconomic status were also significantly associated with place of delivery. Another qualitative study in Tanzania pointed out that bad delivery care experiences of women undermine the reputation of the health care system, lowers community expectations of facility delivery, and sustain high rates of home deliveries [19]. In Sekela district in Ethiopia, one study reported reasons for home delivery as closer attention from family members and relatives, unexpected labour, not being sick at the time of delivery, and family influence [20]. It was further found in the same study that residence, ANC visit during last pregnancy, maternal education level, and knowledge of pregnancy and delivery services had significant association with institutional delivery service utilization.

As most maternal deaths occur during childbirth, presence of trained and equipped medical staff could substantially reduce the risk [21]. This is possible only if deliveries occur at health facilities, thus meriting the need to understanding factors that influence institutional delivery. Although such factors are clearly documented elsewhere, e.g. [15-20], a comprehensive review is lacking. Most studies have assessed place of delivery relying on individual factors (demand side factors) with limited account of systemic factors (supply side factors). Service utilization is a function of both demand and supply factors. The extent of influence of health services received during pregnancy (ANC) on place of delivery is scarce in the literature. Other factors such as inter-spousal discussion about family planning, household headship, and number of times a woman received ANC services during pregnancy have received limited attention as well. This study considers both individual and some systemic factors in the assessment of place of delivery. The objectives are (1) to estimate the proportion of women that delivered at health facilities in the two years prior to the survey, (2) to assess the relationship between quality of ANC and place of delivery, (3) to test a hypothesis that socioeconomic status is not associated with place of delivery, assuming that the user‒fee exemption policy for maternal and child health services in Tanzania was successful, and (4) to identify other factors associated with institutional delivery among women of reproductive age in Rufiji, Kilombero, and Ulanga districts of Tanzania.

## Methods

### Data source, study area, and study design

Data for this study come from a cross-sectional household survey that was conducted in 2011 in Rufiji, Kilombero, and Ulanga districts of Tanzania using two existing and ongoing Health and Demographic Surveillance System (HDSS) platforms, namely, Rufiji HDSS located in Rufiji district, and Ifakara HDSS which occupies portions of Kilombero and Ulanga districts. The data were collected using a closed-ended questionnaire and sought to obtain information on health seeking behaviors and service utilization patterns by women and children of less than five years of age. The main purpose of the survey was to provide baseline estimates for the *Connect* Project, which is currently being implemented in the three districts using the HDSS platforms. More details about the *Connect* Project can be found in [22,23] and [24]. In brief, *Connect* Project tests the hypothesis that introducing a new cadre of paid community health worker, known as Community Health Agent (CHA), into the system, with the necessary supporting operations, including improvement of emergency referral, reduces child mortality, including newborn mortality, improves key maternal health outcomes, and thus accelerates progress towards (or beyond) Millennium Development Goals 4 and 5. Since the HDSS platforms are longitudinal, population-based health and vital events registration systems which monitor demographic events such as births, deaths, pregnancies, and migrations of the individuals in the study area, they were envisioned as suitable forms to monitor the outcomes and impact of the CHAs.

### Sampling and study population

Households for the main survey were selected randomly from a list of all households (sampling frame) under surveillance by the Rufiji, and Ifakara HDSS. Selection of these households was accomplished using probability proportional to size (PPS) technique. Since these households came from villages with unequal number of households, PPS was the ideal method to use to ensure that each village is represented in the sample. In each of the households sampled, all women of reproductive age (15‒49 years) were eligible for interviews. A woman over 49 years of age was interviewed only if she wholly took care of at least one child less than five years of age in order to obtain information on health and health service utilization pattern for the child. The current analysis focused on 915 women of reproductive age whose last birth occurred in the two years prior to the survey. Therefore, data pertaining to this population were extracted from the parent database for analysis to answer the current research questions.

### Variables

The outcome variable was place of delivery for births that occurred in the two years prior to the survey. This variable was binary, with one category for health facility or institutional delivery and the other for non-facility delivery. Non-facility delivery referred to all births that occurred at home, in farms or on a way to a facility/birth before arrival. Institutional delivery was coded as ‘1’ and non-facility delivery was coded as ‘0’ for computational reasons.

Several explanatory variables were considered. Household socioeconomic status was included, resulting from Principal Component Analysis (PCA) of household assets [25]. Five wealth quintiles were constructed based on ownership of a toilet, toilet type, and source of drinking water. The quintiles ranged from the poorest (Q1) to the wealthiest (Q5) such that the higher the quintile the wealthier the woman’s household. Other assets such as household building material were unfortunately unavailable for the PCA. Other explanatory variables included were education, age, marital status, ethnicity, religion, gravidity, pregnancy intentions, district of residence and type of residence (rural or urban). Gravidity was considered a proxy for fertility. Household headship, inter-spousal communication or discussion about family planning matters were also included in the analysis.

Moreover, we included ANC score of 15 health services whose utilization status during pregnancy was available. Each of these had a ‘yes’ or ‘no’ response to whether a woman was weighed, had her blood pressure measured, height measured, urine sampled, blood sampled, abdomen measured, heart rate of the baby assessed, given an injection in the arm to prevent tetanus (TT), counseled on financial preparation for delivery, counseled on breastfeeding immediately after delivery, counseled on danger signs during delivery, counseled on family planning, counseled on identifying emergency transport options, counseled on danger signs of pregnancy, and counseled and tested for HIV. The scores ranged from 0, if a woman received none of the services, to 15 if she received all of these eservices. We assumed that the bigger the score the better the quality of ANC. The other variable included was the number of ANC visits a woman made during pregnancy.

### Statistical analyses

The sample was first analyzed descriptively to obtain frequency distribution of the women across several characteristics. Bivariate analysis was then conducted by cross-tabulating place of delivery against each of the explanatory variables. The explanatory variables were categorical, and those which were not were categorized and therefore the degree of association between each pair of variables cross-tabulated was tested using Chi-Square (χ^2^). Multivariate analysis was performed using logistic regression to assess factors associated with institutional delivery. Beforehand, an assessment of clustering at household level was carried out to check whether the assumption of independence of observations holds. This was prompted by the fact that during data collection, the interview included all eligible women from the same household for households which had more than one. The assumption was that women from the same household may have the same or similar health behaviours. The assessment ultimately showed that the observations were independent of one another because there was no significant evidence of clustering at household level. In performing the logistic regression analysis, a variable was retained in the multivariate model if the log likelihood ratio test showed that its presence improved the overall model [26]. In this case, ANC score of the ANC services was treated as a continuous variable in order to optimize its predictive power. The level of significance was set at 5%. The entire process of data analysis was carried out using STATA (version 11) statistical software.

### Ethical consideration

Ethical approval for the main survey was granted by the Medical Research Coordinating Committee (MRCC) of the National Institute for Medical Research (NIMR) in Tanzania. During the survey, participation was voluntary and each woman signed (or provided a thumb print if she was illiterate) a statement of an informed consent after which she was interviewed. For legal reasons, an assent was sought for participants less than 18 years of age. Data storage and processing were all handled securely within the Ifakara Health Institute where the *Connect* Project is based.

## Results

### Background characteristics

**Figure 1:**
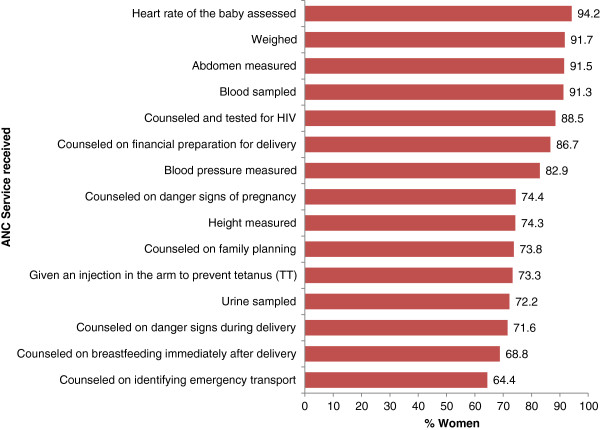
**ANC services received during pregnancy by women who gave birth in two years preceding the survey in Rufiji, Kilombero, and Ulanga districts of Tanzania, 2011 (n** = **907).**

Table 1 shows the distribution of the sample population by maternal characteristics. The mean age of all respondents (n = 915) was 27.9 ± 7.6 years, ranging from 15 to 49. Slightly more than a half (52.0%) of the women were in the first (Q1 ‒ poorest) and second (Q2) socioeconomic quintiles. Women who were in the middle (Q3), fourth (Q4), and fifth (Q5 ‒ wealthiest) quintiles of socioeconomic status were 18.7%, 16.9% and 12.5% respectively. These differences came up because the PCA was performed for all 3,127 women surveyed, but only 915 of them were eligible for the current analysis. Almost 8 out of 10 women (79.8%) were married or living with a partner. While three-quarters (75.5%) of the respondents had at least primary education, the rest (24.5%) had never been to school. Ability to read a letter or newspaper in *Swahili*, Tanzania’s national language, was also assessed and found that 56.4% of the women could easily read, while 15.0% could do so with difficulty, and 28.6% could not read at all. The sample was made up of women from different ethnic groups as *Ndengereko*, 15.3%; *Ngindo*, 14.5%; *Pogoro*, 14.4%; *Sukuma*, 11.4%; and Others, 44.5%. Figure 1 displays the proportion of women that received various ANC services during pregnancy. Results reveal that receipt of the 15 ANC services ranged from 64.4% for counseling on identifying emergency transport to 94.2% for having had the heart rate of the baby assessed.

**Table 1:** **Sample characteristics of women delivered in the two years preceding the survey in Rufiji**, **Kilombero**, **and Ulanga districts of Tanzania**, **2011 (n** = **915)**

**Characteristics**	**Number of respondents (n)**	**Percent (%)**
**TOTAL**	**915**	**100.0**
**Age (in years)**		
<20	135	14.8
20‒34	560	61.2
>34	220	24.0
Mean = 27.9 (SD = 7.6)		
**Marital status**		
Married	729	79.8
Ever married	52	5.7
Single	133	14.6
**Gravidity**		
1	186	20.3
2‒4	429	46.9
>4	300	32.8
Median = 3, IQR = 3	‒‒	‒‒
**Education**		
No formal education	224	24.5
Primary/higher	691	75.5
**Ability to read a letter or newspaper**		
Not at all	261	28.6
With difficulty	137	15.0
Easily	514	56.4
**Ethnicity**		
Pogoro	131	14.4
Ndengereko	140	15.3
Ngindo	132	14.5
Sukuma	104	11.4
Others^‡^	406	44.5
**Religion**		
Christian	389	42.5
Muslim	467	51.0
Other	59	6.5
**District**		
Kilombero	535	58.5
Rufiji	226	24.7
Ulanga	154	16.8
**Type of residence**		
Urban	173	19.0
Rural	740	81.0
**Wealth quintile**		
Q1 (Poorest)	213	25.5
Q2	221	26.5
Q3	156	18.7
Q4	141	16.9
Q5 (Wealthiest)	104	12.5

Number of respondents across some variable categories may not add up to 915 due to some missing data; *SD* = Standard deviation; *IQR* = Inter‒quartile range; ^‡^Small ethnic groups were combined.

### Facility delivery by maternal characteristics

**Figure 2:**
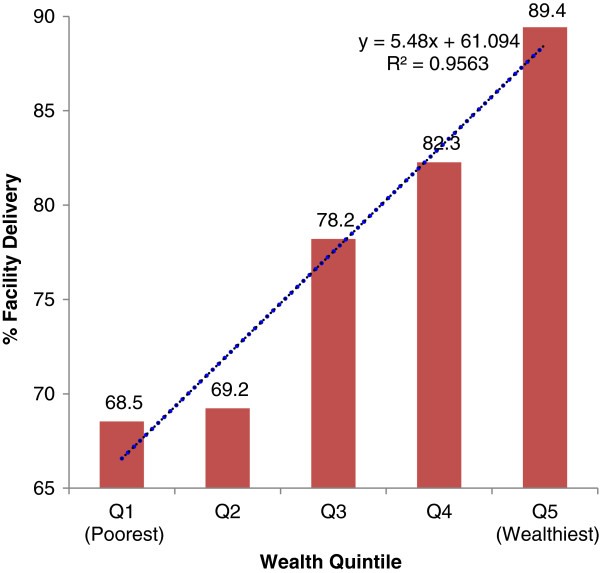
**Percent distribution of institutional delivery by wealth quintiles in Rufiji, Kilombero, and Ulanga districts of Tanzania, 2011 (n** = **835).**

Figure 2, Table 2 and Table 3 show bivariate analyses of place of delivery by each of the explanatory variables. The results reveal that the overall facility or institutional delivery in the two years prior to the survey stood at 74.5% (Table 2), and was as low as 68.5% among the poorest and as high as 89.4% among the wealthiest women (Figure 2). The differences were statistically significant (p < 0.001). In terms of education, facility delivery was 77.6% among women who had primary or higher education and 65.2% among women who had no formal education (p < 0.001). Additionally, 81.5% of the women who were easily capable of reading a letter or newspaper in *Swahili* (Tanzania’s national language) delivered at health facilities. This proportion was 69.3% and 64.4% among women who could do so with difficulty and not at all respectively. The differences were statistically significant (p < 0.001). The extent of institutional delivery did not change significantly by pregnancy intentions (p = 0.631). It was 74.3%, 76.2%, and 78.1% among women who reported having had their pregnancies intended, mistimed, and unwanted, respectively.

**Table 2:** **Percent distribution of women delivered at health facilities in the two years preceding the survey by maternal characteristics in Rufiji**, **Kilombero**, **and Ulanga districts of Tanzania**, **2011 (n** = **915)**

**Characteristics**	**% facility delivery**	**P-Value**
**OVERALL**	**74.5**	
**Age (in years)**		
<20	77.8	0.628
20‒34	73.8	
>34	74.5	
**Marital status**		
Married	73.0	0.060
Ever married	75.0	
Single	82.7	
**Gravidity**		
1	83.3	0.005
2‒4	73.7	
>4	70.3	
**Education**		
No formal education	65.2	<0.001
Primary/higher	77.6	
**Ability to read a letter or newspaper**		
Not at all	64.4	<0.001
With difficulty	69.3	
Easily	81.5	
**Ethnicity**		
Pogoro	84.0	
Ndengereko	79.3	<0.001
Ngindo	72.0	
Sukuma	49.0	
Other	77.1	
**Religion**		
Christian	74.8	<0.001
Muslim	78.6	
Other	40.7	
**Pregnancy intentions**		
Intended	74.3	0.631
Mistimed	76.2	
Unwanted	78.1	
**District of residence**		
Kilombero	73.5	0.510
Rufiji	77.4	
Ulanga	74.0	
**Type of residence**		
Urban	85.6	<0.001
Rural	71.9	
**Respondent is household head**		
No	74.5	0.951
Yes	75.0	
**Number of ANC visits made**		
<4	73.4	0.229
≥4	76.9	
**Inter**‒**spousal discussion about family planning**		
Has never happened	71.0	0.003
Has been happening	79.8	

**Table 3:** **Institutional delivery by each of the antenatal services received among women who delivered in two years preceding the survey in Rufiji**, **Kilombero**, **and Ulanga districts of Tanzania**, **2011 (n** = 907*)

**ANC service**	**Response**	**% Facility delivery**	**P-Value**
Counseled on identifying emergency transport	Yes	77.2	0.027
	No	70.6	
Counseled on breastfeeding immediately after delivery	Yes	78.0	0.001
	No	67.8	
Counseled on danger signs during delivery	Yes	77.4	0.006
	No	68.6	
Urine sampled	Yes	79.5	<0.001
	No	62.7	
Given an injection in the arm to prevent tetanus (TT)	Yes	76.7	0.023
	No	69.3	
Counseled on family planning	Yes	76.7	0.034
	No	69.8	
Height measured	Yes	77.6	0.001
	No	67.0	
Counseled on danger signs of pregnancy	Yes	78.4	<0.001
	No	64.7	
Blood pressure measured	Yes	77.7	<0.001
	No	61.3	
Counseled on financial preparation for delivery	Yes	76.0	0.053
	No	67.8	
Counseled and tested for HIV	Yes	78.0	<0.001
	No	50.5	
Blood sampled	Yes	76.6	<0.001
	No	57.0	
Abdomen measured	Yes	75.5	0.121
	No	67.5	
Weighed	Yes	75.4	0.249
	No	69.3	
Heart rate of the baby assessed	Yes	76.6	<0.001
	No	47.2	

*These questions were asked to 907 women that sought ANC at least once during pregnancy.

The results also show that institutional delivery was highest (82.7%) among single women and lowest at 73.0% among married women. Multi-gravidity was associated with low facility delivery (p = 0.005) as it was highest (83.3%) among primi‒gravida women and lowest (70.3%) among women who had had more than four pregnancies. Regarding ethnicity, facility delivery ranged from 49.0% among *Sukuma* to 84.0% among *Pogoro* women and the differences were statistically significant (p < 0.001). Moreover, facility delivery was lowest (40.7%) among women following traditional or other unspecified beliefs, 74.8% among Christian and 78.6% among Muslim women (p < 0.001). Institutional delivery was lowest (71.9%) in rural and highest (85.6%) among women who resided in urban areas (p < 0.001). Presence of inter-spousal discussion about family planning was associated with highest proportion of institutional delivery (79.8%). This proportion was 71.0% among women who reported absence of inter-spousal discussion about family planning and the difference was statistically significant (p = 0.003).

Table 3 shows institutional delivery by each of the 15 ANC services received during pregnancy. Results show generally that the proportion of women who delivered at health facilities was significantly higher among women who received each one of the services than that observed among women who did not receive the services. Only three of these services ‒ financial preparation for delivery, abdomen measurement, and weight ‒ were not significant at 5% but showed the same direction of the effect.

### Multivariate analysis

Table 4 presents findings from the multivariate analysis that assesses the independent effect of each explanatory variable on institutional delivery. Results show a remarkable influence of the quality of ANC to the access to institutional delivery care. The odds of having had delivered at a health facility increased significantly by 11% for every one ANC received during pregnancy (OR = 1.11, 95% CI: 1.04, 1.18). With respect to socioeconomic status, women from wealthiest (Q5) households were significantly more than 3 times more likely than the poorest (Q1) women to have had delivered at health facilities (OR = 3.24, 95% CI: 1.40, 7.48). The rest of the quintiles, Q4, Q3 and Q2, were not significantly different from Q1 (P > 0.05).

**Table 4:** **Multivariate logistic regression of factors associated with institutional delivery among women that delivered in two years preceding the survey in Rufiji**, **Kilombero**, **and Ulanga districts of Tanzania**, **2011 (n** = **812)**

**Covariate**	**Odds Ratio (OR)**	**95% Confidence Interval (CI)**
**ANC score**^**†**^	1.11***	1.04—1.18
**Wealth Quintile**		
Q1 (reference)	1.00	—
Q2	0.95	0.59—1.56
Q3	1.23	0.69—2.19
Q4	1.82*	0.96—3.46
Q5 (Wealthiest)	3.24***	1.40—7.48
**Gravidity**		
1 pregnancy (reference)	1.00	—
2—4 pregnancies	0.65	0.38—1.09
>4 pregnancies	0.66	0.38—1.16
**Ability to read a letter or newspaper**		
Not at all (reference)	1.00	—
With difficulty	1.11	0.65—1.89
Easily	1.48*	0.96—2.27
**Marital status**		
Married (reference)	1.00	—
Ever married	1.04	0.48—2.25
Single	1.31	0.73—2.34
**Ethnicity**		
Pogoro (reference)	1.00	—
Ndengereko	0.97	0.40—2.33
Ngindo	0.59	0.29—1.19
Sukuma	0.40**	0.16—0.96
Others	0.88	0.49—1.59
**Religion**		
Muslim (reference)	1.00	—
Christian	0.63**	0.40—0.995
Other	0.47	0.15—1.40
**District of residence**		
Kilombero (reference)	1.00	—
Rufiji	1.42	0.71—2.83
Ulanga	3.02***	1.62—5.61
**Urban residence:** (ref = rural)	1.44	0.83—2.51
**Presence of Inter**-**spousal discussion about family planning:** (ref = absence)	1.72***	1.17—2.52

***P < 0.01, **P < 0.05, *P < 0.10; Goodness-of-fit test, P = 0.204; ^†^ANC score was created based on 15 antenatal services, with a minimum score of 0 if no service was received and a maximum of 15 if all services were received (see Figure 1 and Table 3 for details of the services).

Ethnicity was another variable which exerted a significant influence to the access to institutional delivery care. Women from the *Sukuma* ethnic group were 60% less likely than women from the *Pogoro* ethnic group to have delivered at health facilities (OR = 0.40, 95% CI: 0.16, 0.96). Other ethnic groups were not significantly different from the *Pogoro* ethnic group (p > 0.05). Furthermore, facility delivery was 1.72 times more likely among women who reported presence of inter-spousal discussion about family planning compared with those who reported absence of it (OR = 1.72, 95% CI: 1.17, 2.52). The results showed further that women residing in Ulanga district were three times as likely as those in Kilombero district to have delivered at health facilities (OR = 3.02, 95% CI: 1.62, 5.61). Christian women were 37% less likely than Muslin women to have delivered at health facilities (OR = 0.63, 95% CI: 0.43, 0.995).

Finally, at the 5% significance level, other covariates ‒ gravidity, marital status, ability to read a letter or newspaper, place of residence ‒ were not significantly associated with institutional delivery in the multivariate analysis.

### Reasons for non‒facility delivery

**Figure 3:**
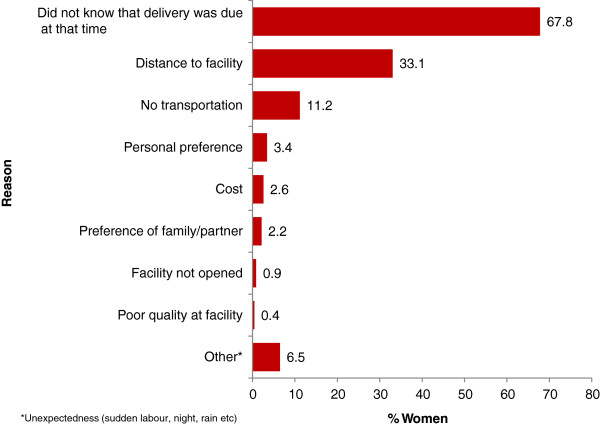
**Reasons for non-institutional delivery and the percent of women reported each reason in Rufiji, Kilombero, and Ulanga districts of Tanzania, 2011 (n = 233).**

Women who did not deliver at health facilities (n = 233) were asked to state their reasons for that. Results in Figure 3 show that two-thirds (67.8%) of these women did not deliver at health facilities because they did not know that they were going to deliver at that time (i.e. sudden onset of labour). Also three cost-related reasons for non-facility delivery were reported: distance to a health facility (33.1%), lack of transportation (11.2%), and costs (2.6%). Other reasons such as woman’s preference, poor quality of care at the facilities and facility not opened were rare with less than 3% of the women reporting each (Figure 3).

## Discussion

This study sought to examine factors affecting access to institutional delivery care, using cross-sectional household survey data collected in Rufiji, Kilombero and Ulanga districts of Tanzania in 2011. Generally, results show that facility delivery was high in the study area, with about three-quarters of the women who delivered in the two years prior to the survey reporting access to institutional delivery care. This proportion was higher than the national estimate from the 2010 Tanzania’s DHS report [13] as well as those of most less developed countries [3,17,20]. Tanzania’s Morogoro region which contains both Kilombero and Ulanga districts had an institutional delivery of 58.0% and so was 73.1% for Pwani region which contains the Rufiji district [13]. While it is clear that this finding may not be regionally or nationally representative, it underscores the reported variations in the proportion of women delivering at health facilities between regions in Mainland, Tanzania, which range from 30% in Rukwa to 90% in Dar es Salaam [13].

Also the results lead into a rejection of the hypothesis that socioeconomic status is not associated with place of delivery, assuming that the user-fee exemption policy was successful. The results showed instead that better socioeconomic status was significantly associated with institutional delivery, implying that the user-fee exemption policy for maternal and child health services may have been less successful in making access to institutional delivery care equitable between the poor and the least poor. This finding is consistent with the 2010 Tanzania’s DHS report [13] and other studies [17,18]. Disregarding the Tanzania’s policy on user fees for maternal and child health services, this observation was expected as better socioeconomic status will enable women overcome their socioeconomic barriers to facility delivery, such as distance, transportation and other associated costs. This suggests probably that women’s understanding of facility delivery is that of a service to be paid for out of pocket and therefore the need to empower women socioeconomically in order to enhance their access to better health care sources for better health outcomes.

Significant ethnic variations in the access to institutional delivery care were observed in the study area, with *Sukuma* ethnic group being less likely than *Pogoro* to deliver at health facilities. This observation signals cultural differences in perceptions, beliefs and practices in various maternal and child health aspects. Similar observation was made by other studies [15,16]. It is possible that some cultures may be strict to their ritual observance than others, thus likely to differ in responding to modern health care. For instance, *Sukuma*, the largest ethnic group in Tanzania originating in the regions of Mwanza, Tabora, and Shinyanga [27], is characterized by songs and dances performed during ritual ceremonies for childbirth, death and work [28]. This may possibly limit institutional delivery in favor of home delivery where these rituals and ceremonies can be organized. Historically, living arrangements of the *Sukuma* people were largely agro–pastoralism and ownership of large cattle herds [27]. In this context, it is possible that some of them may have limited exposure to better information about, and access to modern and recommended care practices such as institutional delivery. This agrees with another study which found that there was a late and underutilization of ANC among the *Sukuma* women due to their distant settlements and lack of education [29].

Furthermore, the significance of the quality of ANC in the access to institutional delivery care suggests, among others, that health institutions have an imperative role to play towards increasing the rate of institutional delivery care utilization through enhancing the quality of and access to antenatal care including such elements as improved staff attitude, counseling, care, and care delivery modalities. If proper ANC is sought, the resulting awareness of pregnancy-associated risks and consequences, and the services received are likely to encourage women to seek appropriate delivery care sources, hence improve pregnancy outcomes. This is important and should be promoted. Therefore, both the demand side and supply side are critical when it comes to institutional delivery care utilization. A similar study in rural northern Ghana found presence of a relationship between ANC and place of delivery [15].

The presence of inter-spousal discussion about family planning was significantly associated with higher chances for institutional delivery care utilization. It is likely that as couples discuss and plan their fertility trajectories, they also consider health facilities as best sources of delivery care to ensure safety and pleasant outcomes. This suggests another educational venue that couples could potentially benefit from as far as discussing and deciding about place of delivery is concerned. Therefore, male partner involvement in fertility planning is very important not only to increasing contraceptive use as observed in previous studies [30-33], but also institutional delivery. So making decisions jointly about delivery and family planning matters should be encouraged between couples to enhance institutional delivery care utilization.

An attempt was made to assess whether pregnancy intentions (intended, mistimed or unwanted) was associated with place of delivery. This factor, according to previous studies, is associated with several health outcomes including poor utilization of ANC and delivery care [34-36], maternal depression [37-41], poor psychological wellbeing [42], anxiety [40], and several others [34,35,43,44]. Therefore we hypothesized that pregnancy intentions are associated with place of delivery in the study area. Our findings, however, did not support the hypothesis because the effect of pregnancy intentions on place of delivery was not statistically significant. Further investigations, possibly with different designs may be relevant to assess whether or not pregnancy intentions are associated with place of delivery in the study area.

Although documented in the literature as predictors of place of delivery, both the number of ANC visits ever made during pregnancy and household headship did not significantly influence institutional delivery care utilization in the study area. The insignificance of the former partly indicates that it may not be just attending, but the type and quality of ANC services received that matter in deciding where to deliver. The latter factor was considered important as far as autonomy is concerned, but likewise had no notable effect on institutional delivery. Similarly, education level was not significant in the multivariate analysis, but ability to easily read a letter or newspaper in *Swahili*, the Tanzania’s national language, became so at borderline (p = 0.075). It is important to note that not only all those who attended any level of school could read, some couldn’t. Ability to read can be an advantage especially with comprehension of written messages such as leaflets, posters, or fliers. These are usually available in health facilities and could positively shape mindsets of women concerning decision making around institutional delivery. Future research could examine among others how the explanatory power of reading skills in various health outcomes compare with that for education, a variable conventionally measured as highest level of education attended or number years spent in school.

Unexpectedly, Christian women were significantly less likely than Muslim women to deliver at health facilities. It was not very clear why this was the case. Therefore, further studies especially qualitative ones may be of relevance to explore any underlying mechanisms behind this observation.

For most of the women who did not deliver at health facilities (n = 233) in the two years prior to the survey, the onset of labour was sudden thereby prompting home delivery as similarly noted in another study [17]. This is the reason two-thirds of the women cited for not having delivered at health facilities. The second and third most reported reasons for not having delivered at health facilities were distance and lack of transport respectively. These have been reported elsewhere [16,17]. While a need for infrastructural improvements is needed, some socioeconomic empowerments are needed as well to enable women overcome socioeconomic barriers, and thus travel to better health care sources for better delivery care.

Finally, the *Connect* Project’s Community Health Agents (CHAs) [24] visit households regularly to provide preventive and some basic curative services in order to accelerate achievement of the Millennium Development Goals (MDGs) 4 and 5 in these three districts of Tanzania. In this process, pregnant women are identified and followed-up, counseled on ANC and related services, and are encouraged to deliver at health facilities for proper care. The CHAs, among others, also promote family planning, postnatal care for the mother and the baby and enrollment to Community Health Fund (CHF) to enhance access to care including delivery care. The *Connect* Project also supports emergency referral and has recently provided ambulance vehicles to the three district councils and two ambulance boats to Rufiji district council. The project further trained health workers including drivers from selected facilities on responding to emergencies including triage, and provided mobile phones to facilities and the CHAs to support communication. Since the CHAs are based in the communities, their phones among others enable CHA-facility communication in case of emergencies, particularly pregnancy- and child-related emergencies.

### Limitations

Distance was reported as one of the barriers to institutional delivery, but was not available for modeling in the multivariate analysis. Also the measure of quality of ANC employed may be inaccurate but was a reasonable proxy within the limits of the data. Generalization of these findings beyond the study districts may be unlikely because only three districts were surveyed. A qualitative study is needed to explore and explain ethnic values, beliefs, and practices concerning childbirth and all issues around it.

## Conclusion

The degree of institutional delivery in Rufiji, Kilombero, and Ulanga districts of Tanzania is high and emerges generally that three in every four pregnant women deliver at health facilities. Improving the quality of ANC is a critical dimension to increasing access to institutional delivery. Socioeconomic empowerment, if viable, is likely to enhance access to better health care and consequently result in better health outcomes. Therefore, interventions should target the poor. Programs aiming at universal access to delivery care should, among others, target vulnerable population groups such as women from the *Sukuma* ethnic group. Moreover, joint discussions and decisions between couples about reproductive and child health matters should be promoted as this also enhances institutional delivery. Overall, pernicious context‒specific conditions should be addressed on the course of realizing universal access to institutional delivery care.

## Competing interests

The authors declare that they have no competing interests.

## Authors’ contributions

AE conceptualized the problem, designed the study, analyzed the data, and drafted and revised different versions of the manuscript. AMK provided assistance with data analysis, study design, and reviewed the manuscript critically. MN, KT, and HVD reviewed the manuscript critically. AH and JFP developed a protocol for the *Connect* Project and reviewed this manuscript critically. All authors read and approved the final draft of the manuscript.
